# A qualitative study assessing how reach and participation can be improved in workplace smoking cessation programs

**DOI:** 10.18332/tpc/161589

**Published:** 2023-03-24

**Authors:** Nikita L. Poole, Gera E. Nagelhout, Tessa Magnée, Lotte C. I. de Haan-Bouma, Cas Barendregt, Onno C. P. van Schayck, Floor A. van den Brand

**Affiliations:** 1 Department of Health Promotion, Care and Public Health Research Institute, Maastricht University, Maastricht, the Netherlands; 2 IVO Research Institute, The Hague, the Netherlands; 3 Erasmus School of Social and Behavioural Sciences, Erasmus University Rotterdam, Rotterdam, the Netherlands; 4 Department of Family Medicine, Care and Public Health Research Institute, Maastricht University, Maastricht, the Netherlands

**Keywords:** workplace smoking cessation, participation, qualitative, employee, implementation

## Abstract

**INTRODUCTION:**

Randomized controlled trials have demonstrated the effectiveness of workplace smoking cessation programs. However, with low participation rates reported, it is important to understand the barriers and facilitators for the reach and participation of employees in workplace smoking cessation programs. The objective of the present study is to uncover the needs of employees regarding reach and participation when implementing a workplace program to address smoking cessation.

**METHODS:**

We carried out 19 semi-structured qualitative interviews in 2019 based on the Reach, Effectiveness, Adoption, Implementation and Maintenance (RE-AIM) Framework with current and former smoking employees of organizations with ≥100 employees in the Netherlands. Some of the interviewees had experience with a cessation program. Data were analyzed using the Framework method.

**RESULTS:**

The main barriers according to employees were insufficient promotion of the cessation program, completing the program in the employee’s own time and working night shifts and peak hours. Facilitators included being actively approached to participate by a colleague, positive reactions from colleagues about employee’s participation in the program, providing the program on location and integrating the program as part of the organization’s vitality policy.

**CONCLUSIONS:**

Effective workplace programs for smoking cessation can stimulate cessation but implementers often experience low participation rates. Our study presents recommendations to improve the recruitment and participation of employees in a workplace smoking cessation program, such as using active communication strategies, training managers to stimulate smoking employees to participate and making the program as accessible as possible by reimbursing time spent and offering the program at the workplace or nearby. Integrating the smoking cessation program into wider company vitality policy will also aid continued provision of the program.

## INTRODUCTION

The workplace is a valuable setting for reaching a large adult population for health promotion programs, such as those that encourage smoking cessation^1,2^. Furthermore, workplace cessation programs are just as effective as those in other settings at stimulating cessation^3^ and effective for those with a lower income and educational level^4-6^, suggesting utility to address smoking in lower socio-economic groups, under which smoking prevalence is often higher^7^. Not only does a smoking employee's health gain from quitting, but employers could also recoup and prevent costs incurred due to increased sick leave or disability, productivity losses owing to smoking breaks and increased healthcare costs^8,9^. Reduced smoking prevalence also translates into lower healthcare costs and increased quality of life years^10,11^.

A problem that remains, however, is the low rate of participation often achieved in such workplace programs^3,12^ which may become progressively more challenging as workers become increasingly mobile due to distance-working^3^, a trend heightened by the COVID-19 pandemic. Part-time or temporary contracts pose additional challenges for recruitment and the scheduling of activities so that they remain accessible to all^13^. Furthermore, there is a dearth of literature regarding the needs of employees when implementing a workplace health promotion program as most of the attention thus far has been given to the employer’s needs.

Workplaces in the Netherlands are moving towards becoming totally smoke-free, with legislation expanding on the 2004 workplace smoking ban^14^ to include the removal of designated smoking rooms in all workplaces in 2022 and introduction of smoke-free outdoor grounds of institutions such as hospitals, mental health facilities and government buildings in 2025^15,16^. With these developments, it would be prudent for employers to consider offering a smoking cessation program to their employees and therefore it is important that we learn how this can be done successfully. In 2019, 26.2% and 24.9% of lower and moderately educated adults in the Netherlands smoked respectively, of which 89.3% and 76.3% smoked daily, compared to 15.4% of highly educated adults who smoked, of which 48.7% smoked daily^17^. To better understand the barriers and facilitators in the reach and participation of employees in workplace cessation programs, we conducted a qualitative needs assessment among employees in the Netherlands, focusing on workplaces with employees with a lower level of education. Our needs assessment focused on the following research questions: 1) ‘How can employees be reached to inform and stimulate them to participate in a smoking cessation program?’, 2) ‘How do colleagues react to the participation of others in the program?’, 3) ‘What are the practical barriers and facilitators to participation in the program?’, and 4) ‘What factors should be considered when maintaining a smoking cessation program in an organization?’.

## METHODS

### Design

Within this qualitative study, we performed individual qualitative interviews (n=19) among employees of organizations in the Netherlands. Interviews were performed between January and June 2019.

### Sample

Purposive sampling was used to recruit current and former smoking employees of organizations with ≥100 employees of which relatively many people have a low level of education. Purposive sampling also made sure a variation of organizations from different sectors and employee occupations were included (Table 1). Our sample included respondents who had and had not experienced a workplace smoking cessation program to explore experienced and anticipated barriers and facilitators to reach and participation. Most of the interviewees were recruited as participants from a previous RCT in which smoking cessation group programs with financial incentives were offered^6^. The smoking cessation program in this RCT consisted of group-based weekly sessions of 1.5 hours for 7 weeks. Interviewees included were from both the treatment group (smoking cessation group program with financial incentives for quit success) and control group (smoking cessation group program without financial incentives). Other interviewees who had not participated in the RCT were recruited via convenience sampling through company representatives. All interviewees received 20€ as compensation for their participation in the interview, which was directly deposited to their bank account. The aims of the research were shared with the interviewees via an information letter and the informed consent form. The Central Committee on Research Involving Human Subjects in the Netherlands requires no ethical approval for non-medical research. The interviewing author and the interviewees did not know each other prior to study commencement.

**Table 1:** Characteristics of interviewees from large Dutch companies (≥100 employees), 2019

*Characteristics*	*n (%)*
**Gender**	
Man	9 (47)
Woman	10 (53)
**Age** (years)	
30–39	5 (26)
40–49	7 (37)
50–59	6 (32)
≥60	1 (5)
**Education level^†^**	
Low	2 (11)
Moderate	11 (58)
High	6 (32)
**Occupation** *	
Managers	3 (16)
Technicians and associate professionals	2 (11)
Clerical support	6 (32)
Services and sales	5 (26)
Plant and machine operators and assemblers	3 (16)
**Sector**	
Education	3 (16)
Emergency services	2 (11)
Financial	1 (5)
Government	3 (16)
Industrial (chemical, horticulture, metal, sheltered work)	5 (26)
Healthcare	1 (5)
Retail	4 (21)
**Participated in cessation program**	
Yes – with financial incentive	11 (58)
Yes – without financial incentive	3 (16)
No	5 (26)
**Current smoker**	
No	11 (58)
Yes	8 (42)

† Education categories – Low: none completed, primary school and lower secondary education; Moderate: middle secondary education; High: upper secondary education and university.

* International Standard Classification of Occupations 2008 (ISCO 2008)

### Data collection

Semi-structured interviews were conducted face-to-face at the workplace by one of the authors (CB), who is trained and experienced in qualitative interviewing. A qualitative approach was used so that employees could share their opinions and experiences in a detailed way, also allowing the interviewer to probe when new or unexpected findings were reported. See the Supplementary file for interview topic lists for employees who have and have not experienced a workplace smoking cessation program.

Interviews lasted between 30 and 80 minutes. Two of the interviews were conducted with a manager or human resources (HR) representative present.

The interview guide was semi-structured and based on the RE-AIM Framework, which stands for Reach, Effectiveness, Adoption, Implementation and Maintenance^18^. We focused on aspects of reach, adoption, implementation and maintenance as the effectiveness of workplace group cessation programs has been investigated in previous work^3,6,19^. For adoption, we asked about the acceptability of the program by employees and their colleagues.

As mentioned, not all interviewees received a workplace smoking cessation program. The cessation program was described to these respondents before asking them what barriers and facilitators they perceived to exist.

### Analysis

All interviews were audio recorded, transcribed verbatim and imported into NVivo 12 (QSR International©, Melbourne, Australia) for coding and analysis. All interviews were coded using the Framework method^20^. This method consists of five stages: 1) familiarization, 2) identifying the thematic framework, 3) indexing, 4) charting, and 5) mapping and interpretation. The first stage, familiarization, was undertaken by CB, LdH-B, TM and NP. CB wrote memos and an overall report of the interviews and LdH-B, TM and NP read the report and the transcripts to familiarize themselves with the data. The report was also shared with the respondents so that they had the opportunity to provide comments. LdH-B and TM completed the second stage (indexing), coding the transcripts both deductively and inductively^21^. The first two transcripts were double coded by LdH-B and TM, before agreeing on a final thematic framework (stage three), whereafter the remaining transcripts were coded individually by LdH-B and TM. Themes were arranged based on the RE-AIM model from which further sub-themes were made, for example, under Reach, sub-themes ‘reasons to participate or quit smoking’ and ‘recruitment general’ were made. These sub-themes were further broken down into individual factors given by the respondents. FvdB and NP created a matrix based on the RE-AIM model and responses were summarized and added to the matrix (stage 4, charting). In the final stage, mapping and interpretation, the matrix was primarily examined by NP for connections and comparisons across respondents within codes, checking the original transcripts for context. Interpretations were discussed within the research team. For only one theme data saturation was not reached, as the sub-theme ‘not suitable for temporary workers’ emerged in the penultimate interview. The data was also analyzed for patterning in responses based on characteristics such as participation in the program, gender and smoking status; however no substantial differences in responses were found.

## RESULTS

### How can employees be reached to inform and stimulate them to participate in a cessation program?

Employees mostly expressed that a more proactive and personal approach is needed to stimulate participation. Respondents felt that more could be done to promote the smoking cessation program as some only received a mass e-mail or saw a message on the intranet (Table 2). They also mentioned that the traditional channels of communication (e-mail, intranet messages) will not necessarily reach all types of employees such as those who are not office-based. A program participant mentioned:

**Table 2:** Barriers and facilitators to the reach and participation of employees in a workplace smoking cessation program

*Barriers*	*Facilitators*
**How can employees be reached to inform and stimulate them to participate in a cessation program?**	
Insufficient promotion of the program, employees not approached personally Being approached to participate with a judgmental tone Shame associated with failing to quit, which prevented talking about participating with colleagues	Being personally approached by team leader/HR staff Hearing about the program from a colleague who would also participate The program being promoted with success stories from past participants Promotional materials available in native languages of employees
**How do colleagues react to the participation of others in the program?**	
Anticipation that colleagues would be negative about time reimbursement Smoking colleagues are skeptical about quit success	Colleagues react positively and supportively to participation Seeing colleagues participate stimulates interest to participate among employees who still smoke Explaining to all employees the purpose of the program and the benefits the program can have for all staff
**What are the practical barriers and facilitators to participation in the program?**	
Having to complete the program in own time or use annual leave Working night shifts and peak hours Program not suitable for temporary workers	Time is reimbursed by the employer The workplace setting lowers the threshold to participate Offering alternatives to a group-based program
**What factors should be considered when maintaining a smoking cessation program in an organization?**	
Program was not long enough for sustained motivation	Enthusiasm for program to be repeated Integrating the program as part of company vitality policy Providing longer aftercare period with follow-up session(s)

*‘I came across it on the intranet, but if I had looked a week later … I could have missed it, the message.’* (Participant 11)

In addition, more proactive promotion of the program would enable more employees to hear about the program:

*‘I actually had to enquire about it myself.’* (Participant 10)

Another barrier mentioned was being approached to participate with a judgmental tone:

*‘Don’t go the usual way of “Yeah, you smoke”, “Our sick leave is so high because you smoke” You know, that blaming.’* (Participant 8)

Respondents reported discussing the announcement of the cessation program with a colleague, which was an important reason as to why they signed up for the program:

*‘At first I was like “no” until my colleague who was super enthusiastic said “ah, we’re going to do that”. That won me over.’* (Participant 2)

A few respondents, however, did not discuss their decision to participate with colleagues, with one employee noting that shame was a barrier to talking about participating with colleagues:

*‘But [not discussing participating with colleagues] has a reason and the reason was the shame that I would feel if I didn’t succeed.’* (Participant 7)

Employees also emphasized the importance of being personally approached, although opinions on whether one should be approached specifically by a direct manager or someone from the HR department varied:

*‘[if you personally approach people] I think employees would feel less like a number. If you receive an e-mail, it says “dear [name]”, but that is of program computer work. If they really visit the smokers, people personally, I think you will achieve more.’* (Participant 6)

A direct manager was thought of by the majority of respondents to be most appropriate as there is more trust built to discuss these topics and because they know which employees smoke, other respondents suggested a member of HR to convey the importance of the program.

Respondents came with their own ideas to promote the program. One respondent who did not participate in the program, from a company with many non-Dutch-speaking employees, recommended that there also needs to be attention for the language in which promotional materials are shared:

*‘It is important that you give a printed sheet in Polish. Give it to the people.’* (Non-participant 3)

Another mentioned that past program participants could be interviewed or share their success stories on the intranet:

*‘How do you make sure that people are still enthusiastic? You only get that with … a few good stories and keep promoting that.’* (Participant 1)

### How do colleagues react to the participation of others in the program?

Colleagues were mostly positive about participation, although less so when the program was not held in the employees’ own time. Respondents mostly reported (anticipated) positive responses from their colleagues about their participation in the program and that they received social support from colleagues for their participation:

*‘My experience is that my colleagues wished me the best.’* (Participant 14)

*‘[my colleagues] think it’s great that I quit. They motivated me to quit.’* (Participant 10)

Seeing colleagues participate in the program also stimulated interest in some smoking colleagues.

Whilst colleagues were typically positive about the respondent’s participation in the cessation program, some wondered whether their colleagues were less positive about their time for the program being reimbursed:

*‘They were positive towards me, but I don’t know what they say behind my back: “how much time does that take?” ’* (Participant 13)

A few respondents said that they had received skeptical reactions from colleagues about how successful they would be at quitting:

*‘Well, there was a bit of “Yeah, (…) you’re not going to make it. You know, you’ve tried so many times, so it won’t work this time either”*.’ (Participant 4)

Some colleagues only shared their skepticism after the program had finished.

### What are the practical barriers and facilitators to participation in the program?

Employees named several practical barriers to participation, spanning the setting and timing of the program. Many respondents emphasized the importance of the program being offered during working hours and not having to use their leave allowance:

*‘I don’t think you should get into that discussion that it’s about hours, because then someone will already drop out. So I think you should fully facilitate it and [the hours] should never be an issue if you ask me.’* (Participant 1)

Respondents who were not reimbursed for their time on the program found this to be a significant barrier to participation:

*‘Our employer let us participate in this, but he has actually said from the start: “We facilitate it in the sense that we make a location available, but you have to invest your own time there”. This has been a thorn in my side, I’m very honest, because I think that if you set up something like this as an employer, which is very good, you should also make people free for that.’* (Participant 2)

The workplace setting also lowered the threshold to participate:

*‘And because it was so accessible, eh, I was already here, it was an hour of your working hours, so the walk was very easy, er, I did it because I thought: well, what have I got to lose? You know, I’m here anyway, I’ll walk over there and I’ll see.’* (Participant 14)

Additional barriers were faced by people who work night shifts or experience peak hours during their shift as the timeslots for the program were sometimes not convenient or were at odds with the demands of work:

*‘I don’t think there are very many people who would like to stay before or after the night shift.’* (Nonparticipant 1)

‘*I think it varies from department to department. I have a peak here from 12:00 to 14:00 (...) After or before that, I can arrange something. But don’t touch my peak, you know?*’ (Non-participant 4)

Lastly, it was reported that it may not be possible to offer the program to all employees, such as those with short, temporary contracts:

*‘The program isn’t for everyone. Some people work for two weeks, sometimes three weeks and then they go again. But this is suitable for permanent workers.’* (Non-participant 3)

### What factors should be considered when maintaining a smoking cessation program in an organization?

Respondents suggested ideas for improving the program and some saw it as part of a wider movement towards a healthier lifestyle. Respondents were happy overall with the program, regardless of abstinence status at the time of the interview. Those who had not successfully quit smoking after the program shared their enthusiasm for it to be repeated for themselves or colleagues who had not yet participated:

*‘I e-mailed [HR] about this: “I actually want to do* [the program] *again, because I didn’t stop then”. Actually yes, I did stop, but then I started again.’* (Participant 12)

The suggestion came from one respondent to integrate the program into existing company vitality policy:

*‘I would perhaps make this a part of a kind of vitality program. It’s not just a healthy diet and a healthier lifestyle, but smoking is part of it too.’* (Participant 9)

Some respondents expressed that, should the program be offered again, they would like the program to be longer than seven weeks, with continued attention paid to quitters for sustained motivation:

*‘… maybe a little more attention, that you also feel the motivation more. If that fades away, you won’t have that incentive anymore.’* (Participant 10)

## DISCUSSION

Our qualitative needs-assessment identified several barriers and facilitating factors in the reach and participation of a workplace smoking cessation program. Firstly, in reaching employees to inform them about the program, many employees felt that their employers’ efforts were not sufficient, especially when employers relied on digital communication (e-mail or intranet messages). Dutch employers also reported that some employees were not reachable through digital communication channels^22^. More generally, passive methods of recruitment for smoking cessation programs, such as public announcements, are associated with lower levels of recruitment and retention^23^. Instead, proactive and personal communication can increase program reach and be particularly beneficial in recruiting those with a low socioeconomic position^24^. In the current study, employees were far more receptive to a more personal approach, with some having decided to participate due to a conversation with a colleague. Rather than relying on incidental word-of-mouth promotion, employers could actively target team leaders or other key figures among their staff to share and promote the program. The introduction of smoke-free policy presents an opportunity to engage with employees on this topic. Although not mentioned by the participants in this study, an additional social aspect that may play a role in the decision to participate is not wanting to lose time with colleagues, which is currently spent on smoking breaks, by quitting^25^.

Not all groups are equally easy to reach, however. In the present study, we found that language ability could be a barrier to reaching all employees, especially where (a large proportion of) employees do not understand or speak the national language(s). With this, it is important to make sure that any materials are translated accurately^26^ and so testing of promotional materials would be advisable. Additionally, in organizations where this is prevalent, word-of-mouth promotion may be particularly beneficial.

Accessibility for low-threshold participation was important to the employees as they felt that the program should be offered on location or nearby, that their time should be reimbursed and fit into their working hours to the extent that that is possible. Our study found that negotiation over employees’ own time investment may deter participation, especially for those who already work unsociable hours. However, some employees suspected that colleagues would regard the reimbursement of hours as unfair, although to what extent this sentiment may have been voiced is unknown. Employers’ views on the program being reimbursed vary, as some recognized the lowered threshold to participation but others felt it was only fair that the employees invested some of their own time^22^. To temper potential negative reactions from colleagues, employers should clearly explain to their employees that by reimbursing time spent on the program, they are fully supporting employees to embark on a healthier lifestyle. Offering a vitality program, under which smoking cessation is one component alongside other topics such as exercise and healthy eating, also gives other employees the opportunity to engage in a healthier lifestyle.

Providing the program at the workplace has been highlighted as an important factor in deciding to participate previously^27^ and was also mentioned as important by the employees in our study. For some occupations, however, simply offering the program on location and reimbursing the time will not be sufficient, as additional barriers are faced by those who experience peak hours (for instance in customer- or client-facing roles) or for employees who work irregular hours, such as night shifts or who are temporary workers. Time-related barriers to participation such as a high workload, inflexibility to leave their immediate work area and competing work obligations have also been reported for other workplace programs^28-31^. Whilst it may not be possible for employees who work from home or who have demanding or incongruous work schedules to participate in a group-based program, these groups should not be forgotten or treated with less priority, as both they and their employer can still benefit from reduced illness and disability as a result of quitting^8^. Temporary workers hold a particularly unstable position in the workplace and may therefore experience more stress^32^, furthering their need for cessation support. Other more flexible options such as telephone, online or individual counselling could be more accessible for these groups. Individual smoking cessation counselling of a similar intensity can be just as effective as group-based counselling^33^ and often requires a smaller time investment from the participant as the sessions are focused on the individual rather than a larger group. This could be offered as an alternative for employees whose work obligations preclude them for joining a group-based program, with the possibility to participate during or after working hours.

Respondents were largely positive about the program, regardless of whether they had remained abstinent at the time of the interview. It is clear that for some, however, further support is needed, be that in the form of a longer program for support or the opportunity to complete the program again. The program was offered to employees as a one-off, whereas Chaiton et al.^34^ estimate that it can take between 6 and 30 serious quit attempts before quitting successfully (for at least one year) and so smoking employees would benefit from being offered the chance to participate in a smoking cessation program more than once. Moreover, communicating this may remove some of the shame in discussing quitting with colleagues.

In order to enable employees to participate more than once, the program would need to be maintained. For the maintenance of the program, a key step would be to entrench smoking cessation support into a greater (existing) health or vitality program offered by the employer. In this way, other barriers can be tackled such as routinising the process of recruitment and delivery of the program^22^. Moreover, establishing a company culture of health promotion through the creation of a comprehensive health or vitality program is a component of program success and sustainability^35^.

More generally within the workplace health promotion field, the attention thus far has been given to the employer’s needs when implementing a workplace health promotion program as program adopters, whilst to our knowledge, the needs of employees have been hardly studied. As such, we uncovered new factors that may hinder or facilitate reach and participation in a workplace health program, such as feelings of shame preventing discussion of the program with colleagues or the provision of promotional materials in other languages. Whilst our study focused on a smoking cessation workplace program, many of the findings may be applicable to other types of health promotion workplace programs in which group training sessions are given. In particular, similar findings regarding time constraints have been reported in other workplace health promotion programs^28-31^. However, the field overall, could benefit from more employee needs assessments being conducted with regard to other health behaviors.

Our study has highlighted some important barriers and facilitators to the recruitment and participation of employees in a workplace group-based smoking cessation program. These factors should be considered and addressed in order to ensure optimal program outcomes. To address some of the main barriers, we present the following recommendations: 1) use pro-active communication strategies such as word-of-mouth to inform about and promote the program, 2) train managers to discuss with and stimulate smoking employees to participate, 3) explain to all employees why the program is being offered and the benefits it can have for all staff, 4) make the program as accessible as possible by reimbursing time spent and offering the program at the workplace or nearby, and 5) integrate the smoking cessation program into wider company vitality policy.

### Strengths and limitations

Strengths of the study include the inclusion of employees from different sectors and occupations, increasing the validity of our results for different workplaces. Secondly, by interviewing employees with and without previous experience with a workplace smoking cessation program, we were able to see whether employees who would be hypothetically recruited for the first time would share the same concerns.

This study is not without methodological limitations. Firstly, none of the employees was from workplaces where a cessation program had been held for more than once; and so we are not able to know which factors might become relevant if the program was run repeatedly. Issues related to reaching and recruiting employees, in particular, may differ as program recruitment becomes more routinised. Whilst our sample included employees from a range of occupational roles and industries, only two participants reported having a low level of education and so certain factors that may be more relevant to this group could have been missed or underreported. Lastly, we recognize that the results of the present study may not necessarily be applicable to workplaces with younger employees (aged <30 years) or smaller workplaces (<100 employees) due to the characteristics of our sample.

## CONCLUSIONS

Workplace programs for smoking cessation are effective in stimulating cessation but implementers often experienced low participation rates. Our study presents recommendations to improve the recruitment and participation of employees in a workplace smoking cessation program, such as using active communication strategies and making the program as accessible as possible by reimbursing time spent and offering the program at the workplace or nearby. Integrating the smoking cessation program into wider company vitality policy will also aid continued provision of the program.

## Data Availability

The data supporting this research are available from the authors on reasonable request.
