# Supplementary material for: A qualitative study assessing how reach and participation can be improved in workplace smoking cessation programs
Source: Tob Prev Cessat. 2023 Mar 24;9:07. doi: 10.18332/tpc/161589 (PMC10037216; doi:10.18332/tpc/161589)
Supplement: Supplementary file 1 [file TPC-9-07-s1.pdf]

Supplement 1

Interview topic lists: for employees who have and have not experienced a workplace smoking cessation programme.

Topic list - employees who <u>have</u> experienced workplace smoking cessation programme

Introduction interview

- Explanation audio recording, anonymous processing, consent to the retention of interview data
- Explain the purpose of the interview (gathering information for implementation)
- Sign informed consent form

Employee background

- What is your age, education and position? How many days a week do you work?
- What kind of company do you work for?
- Do you smoke (still)? If so, how many cigarettes per day? If not, how long ago did you quit smoking? And how many cigarettes did you smoke per day?
- How many of your (direct?) colleagues smoke approximately?

Smoking cessation programme

General

- Have you followed a smoking cessation programme at work? If so, when?
- Were you satisfied with the programme afterwards? Why or why not?

Reach

- How were you told that you could participate in an smoking cessation programme at work (e.g. via email, or through a colleague)? What do you think of this way of being invited? Would another way have been better? Why?
- How did your (smoking) colleagues react to the offer of the smoking cessation programme?
- What was the most important reason for you to participate in the smoking cessation programme?
- What else played a role in your decision whether or not to participate in the programme? What benefits did participation have for you? And what disadvantages?
- What would help to convince your smoking colleagues to (also) participate in the programme?

Adoption

- How did your colleagues react to your participation in the programme? Did smokers react differently than non-smokers?
- (if applicable) How did your colleagues react to the reward you received for quitting smoking? Did smokers react differently than non-smokers?
- What did you think of the way it was checked that you had smoked? (self-reporting and CO measurement).

Implementation

- Did you follow the programme completely as intended? For example, have you been to all the meetings? Why or why not?
- What did you think of the time investment that was asked of you?
- What did you think of the planning (dates/times) and the location of the programme?
- (if applicable) What did you think of the reward (the voucher) and its value? What kind of reward would be better?
- What tips do you have for the employer if they want to offer smoking cessation programme again?

Maintenance

- Would you participate in smoking cessation programme (still or again) in the future if your employer offered you this (again) (and you would then smoke)? Why or why not? When or when not?

Smoke-free workplace

- What would you think if your employer decided to make your work environment smoke-free (for example, by making the entire site smoke-free)? Are you for or against this? Why? How could this be implemented?

Other

- Do you have anything else to add to the interview?

Topic list - employees who <u>did not</u> experience workplace smoking cessation programme

Introduction interview

- Explanation audio recording, anonymous processing, consent to the retention of interview data
- Explain the purpose of the interview (gathering information for implementation)
- Sign informed consent form

Employee background

- What is your age, education and position? How many days a week do you work?
- What kind of company do you work for?
- Do you smoke (still)? If so, how many cigarettes per day? If not, how long ago did you quit smoking? And how many cigarettes did you smoke per day?
- How many of your (direct?) colleagues smoke approximately?

Smoking cessation programme

General

- What would you think if your employer offered smoking cessation programme?

Impression of smoking cessation programme

The smoking cessation programme is a group programme*, in which you, together with colleagues, stop smoking in 7 meetings of 1.5 hours. After a preparation of two weeks, in the 3rd meeting you will quit together. After that, the* programme *continues and you will be guided by experienced trainers in the difficult weeks. The* programme *takes place at the workplace. It depends on the employer whether you follow the* programme *in your own time or in work time. It also depends on the employer whether the successful quitters get a reward or not. Research shows that giving a reward (in the form of gift cards) for quitting and persevering is more successful than not giving a reward.*

Reach

If <u>no</u> smoking cessation programme is offered in this company

- What's a good way to offer that to you and your colleagues?
  - What is appropriate in your company / department? Why?
- Would your (smoking) colleagues respond to the offer of an smoking cessation programme?
- What would be the most important reason for you to participate in the smoking cessation programme?
- What else played a role in your decision whether or not to participate in the programme?

If a smoking cessation programme <u>had</u> been offered at the workplace but interviewee <u>did not participate</u>.

- What did you think about the fact that smoking cessation programme was offered?
- What was the deciding factor for you not to participate?
  - What other reasons were there for not participating?
- Has anything changed in your opinion or situation since then that makes you look at it differently now?

- What then?

Adoption

- How would your colleagues react if you participated? Would that be different for smokers than non-smokers?
- How would your colleagues react if they heard that you would get a reward if you quit and keep it up? Would that be different for smokers than non-smokers?

Implementation

- What do you think of the time investment that is requested from you (7 x 1.5 hours whether or not your own time)?
- What would be favorable times for you to follow the programme?
- What is a convenient location for you?
- What do you think is a reasonable reward? Cash, receipts? What is a reasonable amount?
- What tips do you have for the employer if they want to offer smoking cessation programme?

Maintenance

- Would you participate in smoking cessation programme in the future if your employer offered it to you (and you smoked)? Why or why not? When or when not?

Smoke-free workplace

- What would you think if your employer decided to make your work environment smoke-free (for example, by making the entire site smoke-free)? Are you for or against this? Why? How could this be implemented?

Other

- Do you have anything else to add to the interview?

© 2023 Poole N. L. et al.
